# A secular variation candidate for IGRF-14 based on core-flow inversion via an ensemble Kalman smoother

**DOI:** 10.1186/s40623-025-02289-4

**Published:** 2025-10-16

**Authors:** Kyle Gwirtz, Terence Sabaka, Weijia Kuang

**Affiliations:** 1 University of Maryland, Baltimore County, Baltimore, USA; 2 Geodesy and Geophysics Lab, NASA Goddard Space Flight Center, Greenbelt, USA

**Keywords:** Geomagnetism, Geodynamo, Secular variation, Ensemble data assimilation, Kalman smoother

## Abstract

We present a candidate mean secular variation (SV) model for the 2025.0$-$2030.0 period. The forecasted SV is produced with a data assimilation (DA) system built around a simple frozen-flux model of the core flow and magnetic field near the core–mantle boundary (CMB). An Ensemble Kalman Filter (EnKF) and smoother (EnKS) are used to assimilate Gauss coefficients from the Kalmag field model, to estimate a core flow which is then used to predict changes in the magnetic field. This forecast methodology is tested against past 5-year periods where it is found to be effective in predicting mean SV, and is superior to an otherwise identical setup using an EnKF alone (no EnKS). The inferred core flow is examined and is seen to exhibit structures consistent with the eccentric gyre and westward drift found in traditional inversions. While this study presents an SV candidate, its secondary purpose is to explore and highlight the potential of the EnKS methodology in understanding the geodynamo. Notably, the EnKS algorithm we use requires no adjoint for the model and can be implemented into already existing EnKF-based systems. The ease of implementation and improvement provided by the EnKS make it a desirable addition to other geomagnetic data assimilation systems, particularly those built around full, 3-D numerical dynamo models, for which the production and maintenance of an adjoint can be challenging.

**Graphical Abstract:**

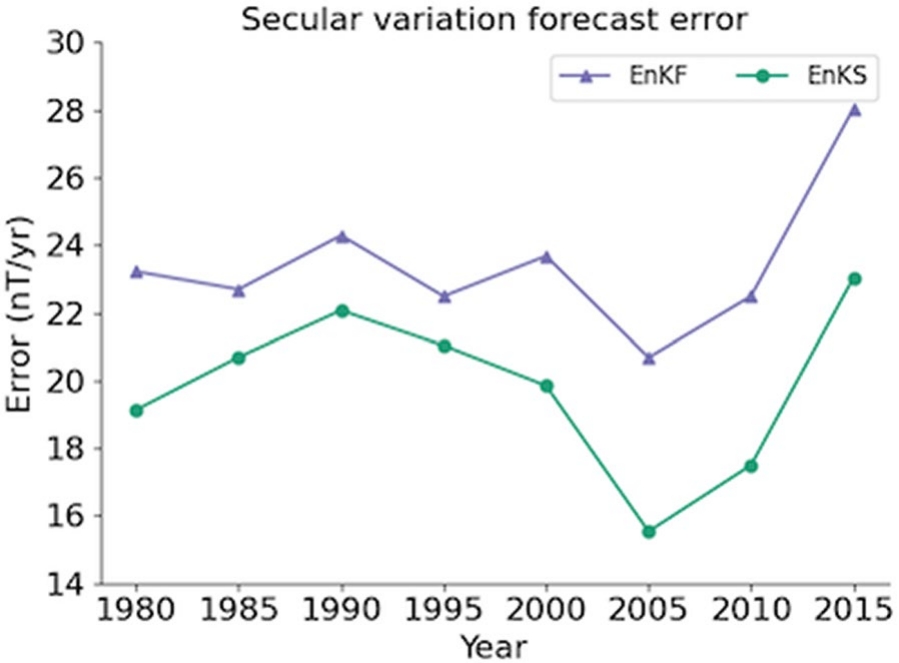

## Introduction

While measurements of the magnetic field around the Earth contain signals from various sources, the majority of the energy in the observed field originates in the Earth’s deep interior (Langel and Estes 1982). This magnetic field is sustained by motion in the electrically conducting fluid of the outer core (geodynamo) and is known to change over time (Jackson et al. 2000; Sabaka et al. 2004, 2020; Baerenzung et al. 2020, 2022; Gillet et al. 2015a; Huder et al. 2020; Cande and Kent 1995; Lowrie and Kent 2004; Ogg 2012). These variations in field morphology provide a unique opportunity to understand the dynamics of the Earth’s deep interior and there have long been efforts to connect magnetic field changes to the fluid flow of the outer core (see, e.g., Roberts and Scott 1965; Whaler 1986; Finlay and Jackson 2003; Lesur et al. 2010; Wardinski and Lesur 2012; Wardinski et al. 2020; Holme 2015; Whaler et al. 2016; Madsen et al. 2025). A technique of growing interest for accomplishing estimates of outer core dynamics is data assimilation (DA, see, e.g., Fournier et al. 2007; Sun et al. 2007; Kuang et al. 2008; Fournier et al. 2010; Tangborn and Kuang 2018; Tangborn et al. 2021; Sanchez et al. 2019, 2020; Minami et al. 2020; Li et al. 2023; Rogers et al. 2025; Suttie et al. 2025). In DA, observations are merged with dynamic models to estimate the true state of a system. The dynamic model, with the state estimate as an initial condition, can then be used to produce forecasts of future changes. Such applications of DA have been a major part of the development of numerical weather prediction over the past several decades (Bauer et al. 2015).

In this paper, we use DA to produce a candidate model for the secular variation (SV) of the main field for the period 2025.0$-$2030.0, as a part of the International Geomagnetic Reference Field (IGRF-14). Past IGRF efforts have included a growing number of DA-based SV candidates (Finlay et al. 2010; Thébault et al. 2015; Alken et al. 2021; Fournier et al. 2021b). While several assimilation systems make use of full 3-D numerical dynamo models (see, e.g., Sanchez et al. 2019, 2020; Fournier et al. 2021a; Tangborn et al. 2021; Aubert 2023), we choose a simplified, frozen flux (FF) model (see, e.g., Roberts and Scott (1965) and Sect. 2.1) with purely toroidal flow. Simplified models have been used before in DA-based field modeling and core flow estimation (Beggan and Whaler 2009, 2010; Barrois et al. 2018; Baerenzung et al. 2018; Ropp et al. 2020; Rogers et al. 2025) and we use this approach because the FF model is computationally inexpensive (compared to a full 3-D numerical dynamo), is relatively easy to experiment with, and is a reasonable approximation over the short timescales considered.

We perform assimilations with the FF model, using an ensemble-based DA approach. An initial ensemble, representing a sampling of the assumed prior at the beginning of an assimilation run, is taken from snapshots of a long run of a full, 3D numerical dynamo model. Specifically, we extract from the dynamo solutions, the components defining the toroidal core flow and poloidal magnetic field near the core–mantle boundary (CMB), which completely describe the state of the FF model (see Sects. 2.1 and 2.4 for details). The assimilations are carried out using an Ensemble Kalman Filter (EnKF, see, e.g., Evensen 2006) combined with an Ensemble Kalman Smoother (EnKS). Kalman smoothers have been used in geomagnetism before, in the production of geomagnetic field models (Ropp et al. 2020; Baerenzung et al. 2020, 2022). With a smoother, the assimilation results can be “smoothed” backwards in time, with the result that state estimates at a given moment can be conditioned on both past and future observations. This differs from the Kalman filter alone, which is causal in that, estimates at a given time are conditioned only on past data. Smoothers have been used in other DA applications to produce assimilation solutions which are more consistent with data than that of a filter alone (Zhu et al. 2003; Khare et al. 2008; Cosme et al. 2010; García-Pintado and Paul 2018; Hasar et al. 2023). In the EnKS we use, the filtering and smoothing are performed iteratively, in a process which under certain conditions, is equivalent to a variational data assimilation approach (4Dvar, see, e.g., (Fisher et al. 2005)). This application of the smoother has the potential to improve forecasts when compared to using just the Kalman filter, because the dynamic model is a nonlinear. Indeed it is well established that in nonlinear DA, the choice of assimilation methodology influences results, and the approach for achieving optimal performance depends on a number of factors including the nonlinearity of the dynamic model, and the nature of the observations (see, e.g., Morzfeld and Hodyss 2019; Metref et al. 2014; Sakov et al. 2012; Bonavita and Lean 2021). Similar to the ensemble variational method of Minami et al. (2020), we choose an approach which avoids the need for determining an adjoint for the model. The production and maintenance of an adjoint can be a demanding task, particularly for complicated numerical geodynamo models. This motivates a secondary purpose of this work, which is to demonstrate the use of the adjoint-free EnKS in the context of core-state estimation, with the intention of seeing it eventually implemented in DA system with a full 3-D numerical dynamo. Indeed the EnKS we use can be implemented outside of any existing EnKF setup, making it appealing for use in established EnKF-based systems such as the Geomagnetic Ensemble Modeling Systems (GEMS, Tangborn et al. 2021; Gwirtz et al. 2024) or pygeodyn (Huder et al. 2019).

To validate the scheme, we produce mean SV forecasts for periods of past IGRF releases which contained a Predictive Geomagnetic Reference Field (PGRF) component. The forecasts are found to compare favorably to past PGRFs and are superior to forecasts produced with the EnKF alone. The method is then used to produce the candidate SV for the 2025.0$-$2030.0 period. Estimates of the core flow are also examined and exhibit evidence of the large gyre and westward drift of traditional core-flow inversions (see, e.g., Finlay et al. 2016; Gillet et al. 2015b; Baerenzung et al. 2018)..

The rest of this paper is organized as follows: Section 2 provides background on the frozen flux model, observations, EnKS, and the scalings needed to relate the observations to the non-dimensional model. In Sect. 3, specific details of steps used to produce forecasts—amount of data to be assimilated, when to perform smoothing iterations—is provided. These steps are used to perform a series of forecast tests which validate the approach over past five year periods. In Sect. 4 we use the procedure to produce the final SV candidate for 2025–2030. The spatial distribution of the predicted SV is examined and the current estimate of the core–surface flow is compared with that of the past periods covered by the validation experiments. This is followed by concluding remarks in Sect. 5

## The dynamic model, observations, and data assimilation methodology

We provide the background details needed to understand the procedure used in producing the candidate secular variation (SV) forecast, and core-flow estimates. First, we discuss the dynamic model (frozen flux with toroidal flow). Then we describe the “observations” (Kalmag, see Baerenzung et al. 2022) and how they are related to the state variables of the dynamic model. Finally, the assimilation procedure (Ensemble Kalman filter and smoother) is presented.

### Simple frozen flux model

The dynamic model model of the assimilation system relies on the frozen-flux approximation, where the radial magnetic field $B_r$ is advected by horizontal fluid flow near the top of the core–mantle boundary (CMB), and magnetic diffusion is neglected. For the production of the candidate SV, we assume purely toroidal fluid flow. Therefore, the fluid flow can be entirely described by a toroidal scalar $\psi$ defining a horizontal fluid flow $\textbf{v}$, just below the CMB, according to

1 $$\begin{aligned} v_{\theta } = \frac{1}{r_m}\frac{1}{\sin \theta }\frac{\partial \psi }{\partial \phi }, \quad v_{\phi } = -\frac{1}{r_m}\frac{\partial \psi }{\partial \theta }, \end{aligned}$$

where $r_m = 3467$ km is the dimensional radius of the model and $v_\theta$ and $v_\phi$ are the polar and azimuthal velocity components, respectively. The flow advects the radial component of the magnetic field $B_r$ according to

2 $$\begin{aligned} \frac{dB_r}{dt} = -(\textbf{v} \cdot \nabla )B_r. \end{aligned}$$

Both the toroidal scalar $\psi$ and the radial magnetic field $B_r$ are defined by spherical harmonic expansions, truncated at degree and order 20:

3 $$\begin{aligned} & B_r = \sum _{\ell =1}^{20}\sum _{m=0}^{\ell } b_\ell ^mY_\ell ^m(\theta ,\phi ) + C.C., \nonumber \\ & \psi = \sum _{\ell =1}^{20}\sum _{m=0}^{\ell } \psi _\ell ^mY_\ell ^m(\theta ,\phi ) + C.C., \end{aligned}$$

where $b_\ell ^m$ and $\psi _\ell ^m$ are the spherical harmonic coefficients of $B_r$ and $\psi$, respectively, $Y_\ell ^m(\theta ,\phi )$ are the (fully normalized) degree $\ell$ and order *m* orthonormal spherical harmonic functions of colatitude $\theta$ and longitude $\phi$, and *C*.*C*. indicates the complex conjugate. Therefore, the state of the dynamic model can be completely described by a single vector

4 $${\mathbf{x}} = (...b_{\ell }^{m} ...\psi _{\ell }^{m} ....)^{T} {\text{ }}$$

containing the spherical harmonic expansion coefficients $b_\ell ^m$ and $\psi _\ell ^m$. The time integration of Equation (2) is performed via a pseudo-spectral method with second-order Runge–Kutta.

It should be noted that the FF model used in this study has several shortcomings. In particular, the model neglects contributions from poloidal flow; is truncated at degree $\ell =20$ and does not resolve or attempt to account for important subgrid-scale processes (see, e.g. Eymin and Hulot 2005; Barrois et al. 2018); and no evolution in time of the velocity field is prescribed, i.e., it remains constant unless adjusted by the assimilation of data, as described below. The original motivation in constructing the model, was to study the EnKS methodology in a low-cost system of minimal complexity, before implementing it with a 3D numerical dynamo model. However, despite the limitations of the simple FF model, it is found to be sufficient for testing purposes *and* the production of short-term forecasts (see section 3). Therefore, no additional effort is made to expand the model in this work. If one did wish to thoroughly investigate inferred core-flow properties or improve forecast quality and range, with the assimilation methodology described below, the authors would encourage the use of a dynamic model of greater complexity (e.g., Huder et al. 2019). In the experiments and conclusions of this study, we highlight some of the potential influences of the simple FF model’s shortcomings.

### Observations to be assimilated

**Fig. 1:**
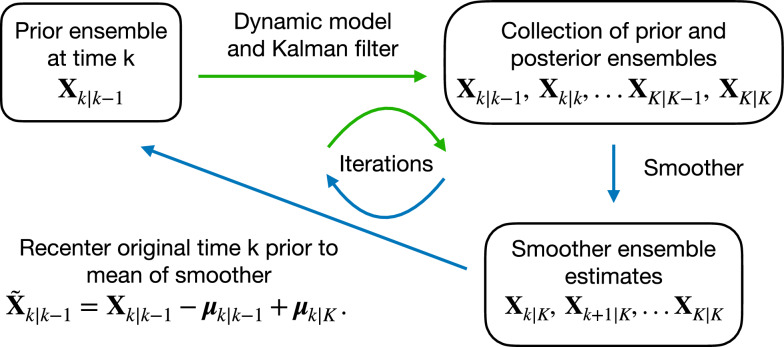
Illustration of the procedure for iterating the ensemble Kalman filter and smoother

**Fig. 2:**
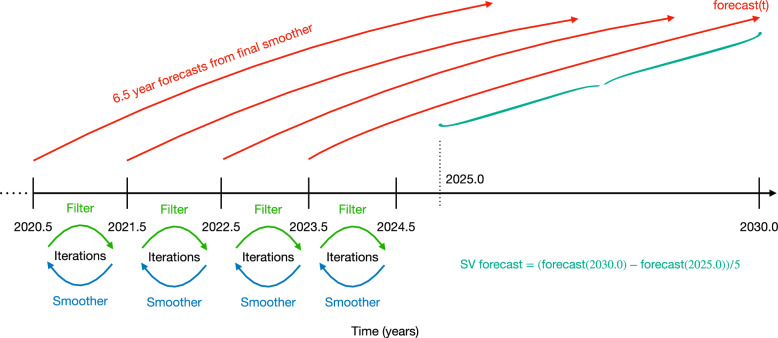
Illustration of the assimilation scheme used in producing the SV forecast. The filtering and smoothing procedure outlined in the text is iterated five times for each pair of observations (separated by one year) before moving to the next pairing of observations, where the process is repeated. At the end of each set of iterations, the smoother ensemble is run forward by the model 6.5 years to produce a forecast. The last such forecast reaches the year 2030.0 and is used to compute the forecasted average SV between 2025.0 and 2030.0

One of the ways DA is unusual in this context is that, rather than dealing directly with observations as one might in numerical weather prediction, spherical harmonic coefficients from field models are used. In this study, we assimilate Gauss coefficients of the core field, through degree 13, from the Kalmag field model (Baerenzung et al. 2022). The coefficients from the midpoint of ten successive years are assimilated (for example, 2015.5, 2016.5, 2017.5,..., 2024.5) with an EnKF and smoothing iterations performed between each pair of assimilation times (see section 2.3 and Figs. 1 and 2). To do this, the Gauss coefficients of the field model must be converted to the complex spherical harmonics coefficients $b_\ell ^m$, of the dynamic model. The Gauss coefficients can first be converted to non-dimensional, “poloidal” field coefficients (see e.g., Gwirtz et al. 2024) according to

5 $$\begin{aligned} P_\ell ^m= \alpha _\ell ^m(g_\ell ^m - ih_\ell ^m), \end{aligned}$$

with

6 $$\begin{aligned} \alpha _\ell ^m=\mathcal {B}_\ell ^m\frac{(-1)^m}{\ell }\sqrt{\frac{2\pi (\delta _0^m+1)}{2\ell + 1}}\left( \frac{r_s}{r_m}\right) ^{\ell +2}, \end{aligned}$$

where $r_s = 6371.2$km, is the mean surface of the Earth, $r_m = 3467$ km is the dimensional radius of the model, $\delta _0^m = 1$ for $m=0$ and $\delta _0^m=0$ otherwise, $g_\ell ^m$ and $h_\ell ^m$ are the Gauss coefficients of the field model, and $\mathcal {B}_\ell ^m$ are fixed scaling factors relating the physical field to the non-dimensional model (see section 2.4). The poloidal field defined by $P_\ell ^m$ can then be converted to the model coefficients (which define the radial component of the field) by an application of the negative of the horizontal laplacian ($- \nabla _{H}^{2}$), which amounts to multiplication by $\ell (\ell +1)$, i.e.,

7 $$\begin{aligned} b_\ell ^m= \ell (\ell + 1)P_\ell ^m. \end{aligned}$$

Note that determining $P_\ell ^m$ is an intermediate step which we only include here for the sake of the observation uncertainty discussion below (Sect. 2.2.1).

#### Observation uncertainties

Data assimilation with an EnKF requires observational uncertainties (see Sect. 2.3). To convert the Gauss coefficient uncertainties of Kalmag, to uncertainties for the model state variables $b_\ell ^m$, Equations (5) and (7) can be applied to $\sigma _{g_\ell ^m}$ and $\sigma _{h_\ell ^m}$ like with $g_\ell ^m$ and $h_\ell ^m$. Specifically, the uncertainties of the real and imaginary parts of $b_\ell ^m$ are $\ell (\ell +1)|\alpha _\ell ^m|\sigma _{g_\ell ^m}$ and $\ell (\ell +1)|\alpha _\ell ^m|\sigma _{h_\ell ^m}$, respectively. In the original validation testing and SV forecast described in Sect. 3.2, in transforming the Kalmag uncertainties, Equation (7) is inadvertently applied as a transform for poloidal field *variances*, meaning the corresponding standard deviations in the radial magnetic field coefficients $b_\ell ^m$ are effectively computed as $\sqrt{\ell (\ell +1)}|\alpha _\ell ^m|\sigma _{g_\ell ^m}$ and $\sqrt{\ell (\ell +1)}|\alpha _\ell ^m|\sigma _{h_\ell ^m}$. The end result is that the Kalmag uncertainties which are used are scaled by a factor of $1/\sqrt{\ell (\ell +1)}$. However, it is found that the forecasts validate better when using the “scaled” uncertainties, than those using the “original” Kalmag uncertainties (see Sect. 3.2). In the results and concluding remarks below, we discuss why this may be the case, and highlight the need for a systematic study of the core-field uncertainties most appropriate for geomagnetic data assimilation. For the remainder of the paper, we use the “scaled” Kalmag uncertainties, with the exception of Fig. 4, where results using the “original” uncertainties are provided for comparison.

### Data assimilation methodology

We provide details of the assimilation methodology (EnKF and EnKS). As several pieces of notation are introduced, we provide a reference Table 2 of Appendix A.

#### Ensemble Kalman Filter

At a time *k*, where an assimilation is to be performed, the “observations” of $b_\ell ^m$ are collected into the vector $\textbf{y}_k$ and are taken to be related to the “true” state of the dynamic model $\textbf{x}^{\text {true}}_k$ according to

8 $$\begin{aligned} \textbf{y}_k = \textbf{Hx}_k^{\text {true}} + \varvec{\eta }_k, \quad \varvec{\eta }_k\sim \mathcal {N}(\textbf{0},\textbf{R}_k), \end{aligned}$$

where $\textbf{H}$ is the observation operator relating the model state to the observations and $\varvec{\eta }_k$ is a Gaussian random variable with mean zero and covariance $\textbf{R}_k$. The observation covariance is taken to be diagonal, with the core-field uncertainties coming from the Kalmag model (see Sect. 2.2.1). The prior estimate of the state at time *k* is given by the *forecast* ensemble $\{\textbf{x}_{k|k-1}^i\}_{i=1}^{N_e}$ of $N_e$ model states, which are conditioned only on data made available through time $k-1$. This ensemble is produced by using the model to propagate forward in time, the ensemble of model states resulting from the last assimilation at time $k-1$. In the case where no previous data have been assimilated, we begin with the initial ensemble as described in section 2.4. The forecast ensemble of prior state estimates is then adjusted to form an *analysis* ensemble $\{\textbf{x}_{k|k}^i\}_{i=1}^{N_e}$, conditioned on the time *k* observations, according to

9 $${\mathbf{x}}_{{k|k}}^{i} = {\mathbf{x}}_{{k|k - 1}}^{i} + {\mathbf{K}}_{k} ({\mathbf{y}}_{k} - {\mathbf{Hx}}_{{k|k - 1}}^{i} + {\text{ }} \in _{k}^{i} ),{\text{ }}{\mathbf{K}}_{k} = {\mathbf{P}}_{{k|k - 1}} {\mathbf{H}}^{T} ({\mathbf{HP}}_{{k|k - 1}} {\mathbf{H}}^{T} + {\mathbf{R}}_{k} )^{{ - 1}} {\text{ }}$$

where $\varvec{\epsilon }^i_k\sim \mathcal {N}(\textbf{0},\textbf{R}_k)$ are observation perturbations added as a part of the stochastic EnKF (Houtekamer and Mitchell 1998), and $\textbf{P}_{k|k-1}$ is the covariance of the forecast ensemble, i.e.,

10 $${\mathbf{P}}_{{k|k - 1}} = \frac{1}{{N_{e} - 1}}\sum\limits_{{i = 1}}^{{N_{e} }} {({\mathbf{x}}_{{k|k - 1}}^{i} - \overline{{\mathbf{x}}} _{{k|k - 1}} )} ({\mathbf{x}}_{{k|k - 1}}^{i} - \overline{{\mathbf{x}}} _{{k|k - 1}} )^{T} T$$

with $\bar{\textbf{x}}_{k|k-1}$ being the forecast ensemble mean. For the validation experiments and SV forecast, we use an ensemble of $N_e=512$ and apply Noise Informed Covariance Estimation (Vishny et al. 2024) to modify $\textbf{P}_{k|k-1}$ and account for sampling error.

#### Ensemble Kalman smoother

Between each pair of assimilation times, we apply an ensemble-based approach to the fixed-lag Rauch–Tung–Striebel (RTS) smoother (see, e.g., Rauch et al. 1965; Raanes 2016). Starting at time *K*, the RTS smoother steps backward in time, adjusting state estimates according to

11 $$\overline{{\mathbf{x}}} _{{k|K}} = \overline{{\mathbf{x}}} _{{k|k}} + {\mathbf{P}}_{{k|k}} {\mathbf{M}}_{k}^{T} T{\mathbf{P}}_{{k + 1|k}}^{{ - 1}} (\overline{{\mathbf{x}}} _{{k + 1|K}} - \overline{{\mathbf{x}}} _{{k + 1|k}} ){\text{ }}$$

where $\textbf{P}_{k\vert k}$ is the posterior covariance (analysis covariance) at time *k*, $\textbf{P}_{k+1\vert k}$ is the prior covariance (forecast covariance) at time $k+1$ and $\textbf{M}_k$ is the dynamic model. Clearly, the dynamic model of Equation (2) is nonlinear in $\textbf{x}$ and therefore directly applying Equation (11) would require the computation of an adjoint. Additionally, Equation (11) requires full covariances/precision matrices (note that for the EnKF, one only requires ${\mathbf{P}}_{{k|k - 1}} {\mathbf{H}}^{T}$). Rather than directly applying Equation (11), we use an ensemble approximation which is simpler to implement and would be more tractable if applied in the future to, e.g., a data assimilation system with a full 3-D numerical dynamo model.

An outline of the justification for the ensemble approximation is as follows. Let $X_{{k|K}}$ be an array of $N_e$ ensemble members (columns) $\textbf{x}^i_{k|K}$, at time *k*, conditioned on data up to time *K* and let $\varvec{\mu }_{k\vert K}$ be an array of $N_e$ identical columns equal to the ensemble mean ($\bar{\textbf{x}}_{k\vert K}$). Then, e.g., ${\mathbf{P}}_{{k + 1|k}} \approx ({\mathbf{X}}_{{k + 1|k}} - \mu _{{k + 1|k}} )({\mathbf{X}}_{{k + 1|k}} - \mu _{{k + 1|k}} )^{T} /(N_{e} - 1)$ and since $(\textbf{X}_{k+1|k} - \varvec{\mu }_{k+1|k}) \approx \textbf{M}_k (\textbf{X}_{k|k} - \varvec{\mu }_{k|k})$ it can be seen that

12 $${\mathbf{P}}_{{k|k}} {\mathbf{M}}_{k}^{T} T{\mathbf{P}}_{{k + 1|k}}^{{ - 1}} \approx ({\mathbf{X}}_{{k|k}} - \mu _{{k|k}} )({\mathbf{X}}_{{k + 1|k}} - \mu _{{k + 1|k}} )^{ + } {\text{ }}$$

where the superscript $^+$ indicates the pseudo-inverse (Penrose 1955). With this approximation, we can recast Equation (11) in terms of ensembles, giving the smoothing procedure:

13 $$\begin{aligned} \textbf{X}_{k\vert K} = \textbf{X}_{k\vert k}+(\textbf{X}_{k\vert k}-\varvec{\mu }_{k\vert k})(\textbf{X}_{k+1\vert k}-\varvec{\mu }_{k+1\vert k})^+(\textbf{X}_{k+1\vert K}-\textbf{X}_{k+1\vert k}). \end{aligned}$$

The full details of the derivation of Equation (13) from Equation (11) can be found in Appendix B. To step back more than one assimilation time with the smoother, one simply applies Equation (13) multiple times. The first application determines $\textbf{X}_{K-1\vert K}$, which is then used to determine $\textbf{X}_{K-2\vert K}$, and so forth.

To iterate the filter and smoother, we start a new EnKF run from time *k*, with an updated ensemble $\tilde{\textbf{X}}_{k\vert k-1}$ from the smoother ensemble. To produce the updated ensemble, the mean of the original, prior ensemble at time *k*, is shifted to that of the smoother, i.e.,

14 $$\begin{aligned} \tilde{\textbf{X}}_{k\vert k-1}=\textbf{X}_{k\vert k-1}-\varvec{\mu }_{k \vert k-1} + \varvec{\mu }_{k\vert K}. \end{aligned}$$

Fig. 1 provides a flowchart for this entire process. Beginning with a prior ensemble at time *k*, apply the dynamic model and EnKF in the usual way, to assimilate data up to time *K*, while storing all prior and posterior ensembles at assimilation times. Then, apply the smoother of Equation (13) to step back through the desired set of assimilation times. To begin a new iteration, shift the mean of the original time *k* prior ensemble, to that of the time *k* smoother ensemble, as in Equation (14).

In the assimilation runs of this paper, we iterate this process five times between each set of two observations (one year apart, see Sect. 3.1 and Fig. 2). Smoothers and variational methods can be used in this way, over subsets or “assimilation windows” of the data, in various configurations (see,e.g., Bocquet and Sakov 2014; Raboudi et al. 2018; Evensen et al. 2024; Bonavita and Lean 2021). In chaotic/nonlinear systems, there is a limit to the size of the assimilation window over which one can effectively apply such methods—the amplification of small perturbations make it infeasible to determine initial conditions which provide a fit to the data beyond a certain timeframe. For the geodynamo, 10 years may be a reasonable choice to treat in one assimilation window *with a sophisticated physical model*. In this study, because the velocity field of the simplified dynamic model is only adjusted by data—we do not prescribe any evolution in time otherwise—we limit ourselves to short, one-year assimilation windows. To smooth over 10 years of data at once with the simple model, one would have to hope it possible to converge on an initial, static flow, which both fits the assimilated data and provides a decent forecast, i.e., a static flow providing a $\sim 15$ year fit to the observed SV. Future studies, using more sophisticated models, will need to explore optimal assimilation window size.

### Initial ensemble and scaling

The initial ensemble represents a sampling of the assumed prior distribution at the beginning of an assimilation run. We extract the initial ensemble from snapshots of a long run of a self-consistent 3-D numerical dynamo simulation. Specifically, we use the $N_e=512$ member, initial ensemble of the $\textbf{M1}$ dynamo model outlined in Gwirtz et al. (2024). While the initial ensemble members of that study consist of a full description of the 3D dynamo model state, including spherical harmonics defining the toroidal and poloidal fluid flow and magnetic field at various radial depths, we use only a subset of that model description. In particular, we take for the FF initial ensemble, only the spherical harmonic coefficients defining the toroidal flow and poloidal magnetic field, through degree and order 20, near the CMB, i.e., the FF model state outlined in Sect. 2.1 and Equation (4). To relate the non-dimensional state of the model to physical values, we determine scaling factors for time and the magnetic field. These scaling factors are determined using the initial ensemble and the Kalmag field model.

To determine the time scale, we consider the velocity field integrated over the model domain

15 $$||{\mathbf{v}}||^{2} = \frac{1}{{4\pi }}\mathop{{\int\!\!\!\!\!\int}\mkern-21mu \bigcirc} {|{\mathbf{v}}|^{2} \sin \theta d\theta d\phi = \frac{1}{{4\pi }}\sum\limits_{{\ell ,m}} \ell (\ell + 1)|\psi _{\ell }^{m} |^{2} ,}$$

where $|\textbf{v}|^2 = v_\theta ^2 + v_\phi ^2$. We compute the mean of $||\textbf{v}||$ among all $N_e=512$ ensemble members and scale time such that $\overline{||\textbf{v}||}=$15 km/yr. This choice is made such that the typical initial velocity is of the same order of magnitude recovered in traditional core-flow inversions. Since the simple FF model does not prescribe an evolution for the core flow (see Sect. 2.1) and it is instead, only adjusted by assimilation, the time scaling’s *only* influence on results is through the starting distribution of the initial ensemble. The assimilation experiments below quickly adjust the flow to a magnitude of $||\textbf{v}|| \sim$ 20 km/yr (see Sect. 4). For the magnetic field, the scaling factor is determined by the mean intensities in the initial ensemble and Kalmag field model. The surface integral of the magnetic field intensity over the model domain is given by:

16 $$||{\mathbf{B}}||^{2} = \frac{1}{{4\pi }}\mathop{{\int\!\!\!\!\!\int}\mkern-21mu \bigcirc} {|{\mathbf{B}}|^{2} \sin \theta d\theta d\phi = \frac{1}{{4\pi }}\sum\limits_{{\ell ,m}} {\frac{{(2 - \delta _{m}^{0} )(2\ell + 1)}}{{\ell + 1}}} |b_{\ell }^{m} |^{2} }$$

We choose to determine separate scaling for the axial dipole (AD, $\ell =1, m=0$) as the strongest component, and the non-axial dipole (NAD) field, due to the differing aspect ratios of AD/NAD for the initial ensemble and the observational record (see Gwirtz et al. 2024). Breaking Equation (16) apart into the components due to the axial dipole $||\textbf{B}_{\text {AD}}||$ and non-axial dipole $||\textbf{B}_{\text {NAD}}||$, and truncating at degree $\ell =13$, we have

17 $$\begin{aligned} & ||\textbf{B}_{\text {AD}}|| = \sqrt{\frac{3}{8\pi }}|b_1^0|, \quad ||\textbf{B}_{\text {NAD}}|| \nonumber \\ & = \sqrt{\frac{3}{4\pi }|b_1^1|^2+\frac{1}{4\pi }\sum _{\ell =2}^{13}\sum _{m=0}^\ell \frac{(2-\delta _m^0)(2\ell +1)}{\ell + 1}|b_\ell ^m|^2}. \end{aligned}$$

We compute the mean value of the quantities of Equation (17) for the 512 ensemble members ($\overline{||\textbf{B}^{ens}_{\text {AD}}||}$, $\overline{||\textbf{B}^{ens}_{\text {NAD}}||}$) and the observations of the Kalmag field model from 1900–2020 ($\overline{||\textbf{B}^{obs}_{\text {AD}}||}$, $\overline{||\textbf{B}^{obs}_{\text {NAD}}||}$). The ratios of these values are then used to determine the magnetic scaling factors:

18 $$\begin{aligned} \mathcal {B}_\ell ^m = {\left\{ \begin{array}{ll} \overline{||\textbf{B}^{ens}_{\text {AD}}||}/\overline{||\textbf{B}^{obs}_{\text {AD}}||} & \ell =1, m=0\\ \overline{||\textbf{B}^{ens}_{\text {NAD}}||}/\overline{||\textbf{B}^{obs}_{\text {NAD}}||} & \text {otherwise}, \end{array}\right. } \end{aligned}$$

which are used in Equation (5).

## Forecast method and validation

We provide the details of the assimilation steps used to produce the SV candidate. A set of experiments are carried out in which the performance of the forecasting method is validated against past, observed SV. The results of the experiments are used to determine the uncertainties which we report for the SV candidate.

### Assimilation and forecast procedure

Figure 2 illustrates the scheme that is used to produce the SV candidate forecast. The Gauss coefficients through degree 13 are assimilated annually from the midpoint of the ten years preceding the forecast period (2015.5, 2016.5, 2017.5,..., 2024.5), and between each pair of observations the filter and smoother are iterated five times (green and blue arrows of Fig. 2). The final (fifth) smoother estimates (at the earlier of each pair of observation times) are then used as initial ensembles/conditions for forecasts (red arrows) produced by running each ensemble member forward for 6.5 years.

Recall from Sect. 2.1 that one of the shortcomings of the simple FF model is that the flow is only adjusted by assimilation—the entirety of each 6.5 forecast assumes a static flow. The choice of 6.5 years is such that the final such forecast from the filter/smoother iterations between 2023.5$-$2024.5 produces a forecast for the year 2030.0. This last 6.5-year forecast is the only one which is used to produce the candidate SV. The forecasts from earlier in assimilation runs are only considered as a part of the validation experiments of section 3.2. It should be noted that at the starting time of each 6.5-year forecast, the initial (smoother) ensemble is conditioned on observations one year in the future and therefore, while we label these as 6.5-year forecasts to indicate then length of time over which the model is run without further assimilation, the forecast covers only 5.5 years of time over which no data has been made available.

To produce an ensemble of SV estimates, Equations (5) and (7) are used to convert the coefficients $b_{\ell ,i}^m$ of the “*ith*” ensemble member of the dynamic model forecasts, to Gauss coefficients $g_{\ell ,i}^m$, $h_{\ell ,i}^m$ at the Earth’s mean surface. The 5-year mean SV forecasts of each ensemble member is then computed as:

19 $$\begin{aligned} & \dot{g}_{\ell ,i}^m = \frac{g_{\ell ,i}^m(2030.0) - g_{\ell ,i}^m(2025.0)}{5}, \nonumber \\ & \dot{h}_{\ell ,i}^m = \frac{h_{\ell ,i}^m(2030.0) - h_{\ell ,i}^m(2025.0)}{5} \end{aligned}$$

for $i=1,...N_e$. For the final candidate SV and in the validation experiments below, we compute the ensemble means

20 $$\begin{aligned} \bar{\dot{g}}_{\ell }^m = \frac{1}{N_e}\sum _{i=1}^{N_e}\dot{g}_{\ell ,i}^m,\quad \bar{\dot{h}}_{\ell }^m = \frac{1}{N_e}\sum _{i=1}^{N_e}\dot{h}_{\ell ,i}^m \end{aligned}$$

and standard deviations

21 $$\begin{aligned} \sigma _{\dot{g}_{\ell }^m} = \frac{1}{N_e-1}\sum _{i=1}^{N_e}(\dot{g}_{\ell ,i}^m-\bar{\dot{g}}_{\ell }^m)^2,\quad \sigma _{\dot{h}_{\ell }^m} = \frac{1}{N_e-1}\sum _{i=1}^{N_e}(\dot{h}_{\ell ,i}^m-\bar{\dot{h}}_{\ell }^m)^2. \end{aligned}$$

### Forecast validation experiments

To validate the method of prediction, we produce SV forecasts for past periods where the PGRF was produced and Definitive Geomagnetic Reference Fields (DGRFs) are available as a measure of the “truth”. For example, we take the average annual change in coefficients between DGRF-2015 and DGRF-2010 to give the “true” $\dot{g}_\ell ^{m,\text {true}}$, $\dot{h}_\ell ^{m,\text {true}}$ for the 2010.0$-$2015.0 period. This is done for eight such periods (of 5 years), starting with 1980–1985 and ending in 2015–2020. The setup is identical to that used for the final 2025–2030 forecast outlined in section 3.1 and Fig. 2. For example, for the 1980–1985 SV forecast, we assimilate observations from 1970.5, 1971.5,...1979.5, while iterating the filter and smoother five times between each pair of observations. The final smoother ensemble estimate (the 1978.5 smoother ensemble from iterations between 1978.5$-$1979.5) is then used to produce a forecast of the SV between 1980–1985, as in Equation (19) and (20). We compute the SV forecast error by degree according to

22 $$\begin{aligned} & \text {Error}^2(\ell ) = \frac{1}{4\pi }{\int\!\!\!\!\!\oint} |\dot{\textbf{B}}_\ell - \dot{\textbf{B}}_\ell ^\text {true}|^2\sin \theta d\theta d\phi \nonumber \\ & =\sum _{m=0}^{\ell }(\ell +1)[(\bar{\dot{g}}_\ell ^{m}-\dot{g}_\ell ^{m,\text {true}})^2 + (\bar{\dot{h}}_\ell ^{m}- \dot{h}_\ell ^{m,\text {true}})^2], \end{aligned}$$

where $\dot{\textbf{B}}_\ell$ and $\dot{\textbf{B}}_\ell ^\text {true}$ are the forecasted and “true” SV, respectively, of the field due to degree $\ell$, and the total error is given by:

23 $$\begin{aligned} \text {Error} = \sqrt{\sum _{\ell =1}^8\text {Error}^2(\ell )}. \end{aligned}$$

**Fig. 3:**
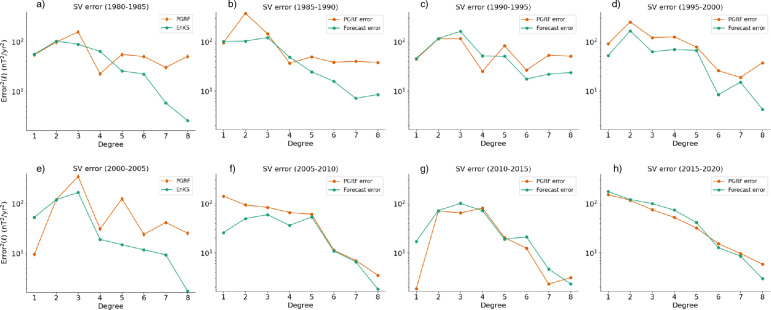
Error in SV, by degree, of PGRF (orange diamonds) and our forecast system (green circles) for the periods of a) 1980–1985; b) 1985–1990; c) 1990–1995; d) 1995–2000; e) 2000–2005; f) 2005–2010; g) 2010–2015; and h) 2015–2020

Figure 3 shows the square error by degree for the PGRF (orange) and forecasting system (green) in each of the eight periods considered.

**Fig. 4:**
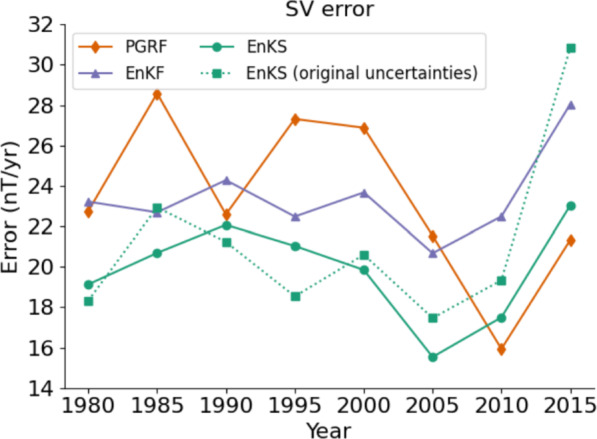
Total errors in forecasted SV, plotted against starting year of the prediction period. For example, the total error in SV predictions for the period 1990–1995 is plotted for the year 1990. Forecast errors are show for PGRFs (orange diamonds), EnKS forecasts (green circles), EnKF only forecasts (purple triangles) and EnKS forecasts with the original Kalmag uncertainties (green squares with dotted line)

Examining the plots, it can be seen that while the degree eight forecast is always better with the forecasting system, there appears to be no other consistent pattern to the scales at which the forecast system outperforms/underperforms the PGRF. The total error, as a function of the beginning of the SV forecast period, is shown in Figure (4), for PGRFs (orange diamonds) and the EnKS forecasts (green circles). Also shown for comparison are the forecast errors using only the EnKF (purple triangles) and the EnKS with the original Kalmag uncertainties (green squares with dotted lines, see Sect. 2.2.1).

Except for the two most recent periods, the overall EnKS SV forecast error is always lower than the PGRF, with an average value of 19.85 nT/yr compared with 23.37 nT/yr for the PGRFs. It is also lower on average than the EnKS with the original Kalmag uncertainties (with a mean error of 21.16 nT/yr), particularly in recent periods, and it is always lower than the EnKF only forecasts (which have a mean error of (23.45 nT/yr).

**Fig. 5:**
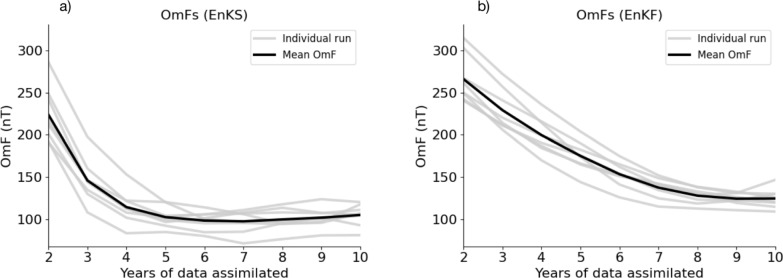
OmFs of forecasts when compared with Kalmag (truncated at degree $\ell =8$), as a function of the number of years that have been assimilated. Each grey line comes from an assimilation run leading up to one of the eight SV validation forecasts. The black line reports the mean OmF. Panel (a) shows results using the EnKS configuration while panel (b) shows results using only the EnKF

To examine whether the 10-year spans of observations are sufficient, we examine the errors of forecasts as progressively more data are being assimilated. For example, during the 1980–1985 SV validation experiment, we produce a 6.5-year forecast, starting from 1970.5 (ending in 1977), after assimilating (with the filter/smoother iterations) 2 years of data (1970.5 and 1971.5). The 6.5-year smoother forecast from 1971.5 (ending in 1978) is conditioned on 3 years of data (1970.5, 1971.5 and 1972.5); the forecast starting in 1972.5, 4 years, and so on. We also similarly produce EnKF only forecasts. For example, during the 1980–1985 SV validation, using only the EnKF, a forecast for 1977 is produced after assimilating 2 years of data (1970.5 and 1971.5); a forecast for 1978 uses 3 years (1970.5, 1971.5 and 1972.5) and so forth. We compute the errors through degree 8, by comparing, not against SV, but with the Gauss coefficients of the Kalmag field model at the end of the forecast. To differentiate from the SV error of the validation experiments, we refer to these values as the “Observations-minus-forecast” errors, or OmFs. They are computed similarly to the SV errors of Equation (22) and (23) but with the Gauss coefficients at the end of the forecast period, rather than the SV, i.e.,

24 $$\begin{aligned} \text {OmF} = \sqrt{\sum _{\ell = 1}^8\sum _{m=0}^{\ell }(\ell +1)[(\bar{g}_\ell ^{m}-g_\ell ^{m,\text {true}})^2 + (\bar{h}_\ell ^{m}- h_\ell ^{m,\text {true}})^2]}, \end{aligned}$$

where $\bar{g}_\ell ^{m}$ and $\bar{h}_\ell ^{m}$ are the mean coefficients of the ensemble at the end of the forecast. Figure 5 shows the OmF of each of the eight runs used in validation (grey) for the EnKS (left panel) and EnKF only (right panel), as a function of the number of years of observations assimilated. The average error is shown in black.

It is seen that after 10 years of data have been assimilated, the forecast errors have converged, with the EnKS producing lower errors than the EnKF alone (as seen in the SV errors of Fig. 4).

It is notable that the forecast errors of the EnKS reach their minimum more quickly than the EnKF alone. The shorter “spin-up" period (time where errors are decreasing) of the EnKS further motivates its consideration with a more complex model. While for the simple FF model, the ten assimilation cycles is sufficient for convergence with the EnKF, estimating the dynamic state with a more informative but more complex model is expected to require a longer time series of observations. For example, results from DA experiments with geodynamo models suggest that for a Kalman Filter, this spin-up period may be on the order of centuries or more (Li et al. 2023; Sanchez et al. 2019; Fournier et al. 2013; Liu et al. 2007). The limited high-quality observational record of the core field therefore makes methods which converge more quickly, particularly desirable.

#### Forecast uncertainties

**Fig. 6:**
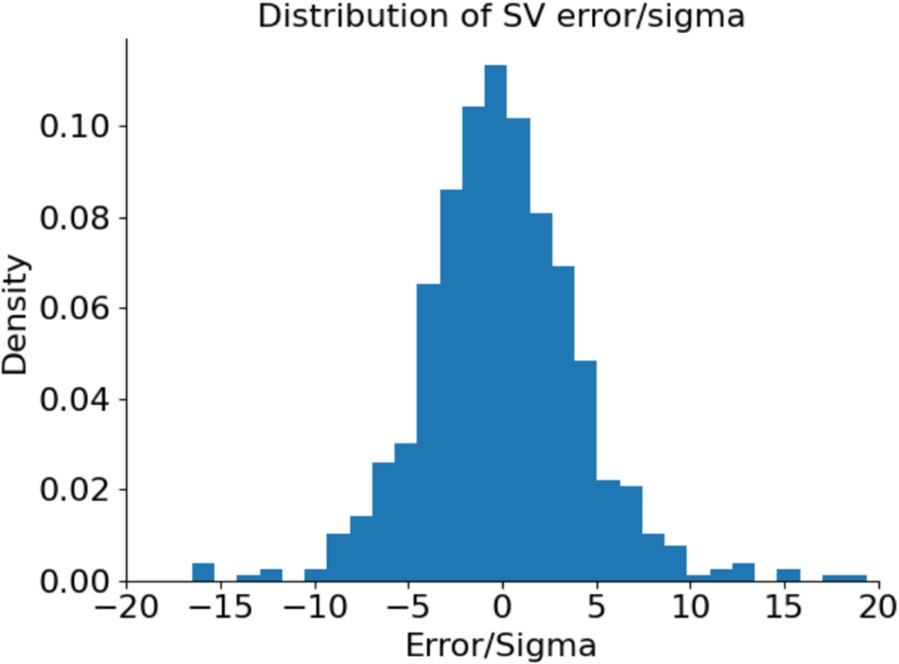
Distribution of the ratios $(\bar{\dot{g}}_\ell ^{m}-\dot{g}_\ell ^{m,\text {true}})/\sigma _{\dot{g}_{\ell }^m}$ and $(\bar{\dot{h}}_\ell ^{m}-\dot{h}_\ell ^{m,\text {true}})/\sigma _{\dot{h}_{\ell }^m}$ for the SV forecasts of coefficients, through degree $\ell =8$, from all eight validation test periods of section 3.2

The SV ensemble uncertainties of Equation (21) cannot account for the errors due to biases such as missing physics in the model. Indeed, even in DA with 3D numerical dynamo models, ensemble standard deviations can be insufficient for capturing the true SV forecast errors (Tangborn et al. 2021; Minami et al. 2020; Fournier et al. 2021a). Therefore, given the shortcomings of the simple FF model (see Sect. 2.1), we also expect this to be the case for the candidate SV of this study. For example, the static flow used for the forecasts likely leads to an underestimation of errors as a result of the ensemble failing to sample potential changes in the flow over the forecast period. To report more useful information about the uncertainties, we examine the relation between magnitudes of $\sigma _{\dot{g}_{\ell }^m}$ and $\sigma _{\dot{h}_{\ell }^m}$, and the size of the “true” errors of the mean ensemble SV, $\bar{\dot{g}}_\ell ^{m}-\dot{g}_\ell ^{m,\text {true}}$ and $\bar{\dot{h}}_\ell ^{m}-\dot{h}_\ell ^{m,\text {true}}$. Figure 6 shows the distribution of the ratios of $(\bar{\dot{g}}_\ell ^{m}-\dot{g}_\ell ^{m,\text {true}})/\sigma _{\dot{g}_{\ell }^m}$ and $(\bar{\dot{h}}_\ell ^{m}-\dot{h}_\ell ^{m,\text {true}})/\sigma _{\dot{h}_{\ell }^m}$ for all coefficients through degree $\ell =8$ of the eight validation periods of the above experiments, for a total of 640 forecasts of SV for individual coefficients.

The errors have a mean near zero, but can be the size of several standard deviations of the ensemble SV ($\sigma _{\dot{g}_{\ell }^m}$ and $\sigma _{\dot{h}_{\ell }^m}$) with the largest outliers being over 15 ensemble standard deviations. For this reason, the uncertainties we report in the candidate SV and in Table 1, are five times the ensemble standard deviation. In the validation experiments of Fig. 6, a five sigma range encompasses 82% of the errors.

## The candidate forecast and inferred core flow

**Fig. 7:**
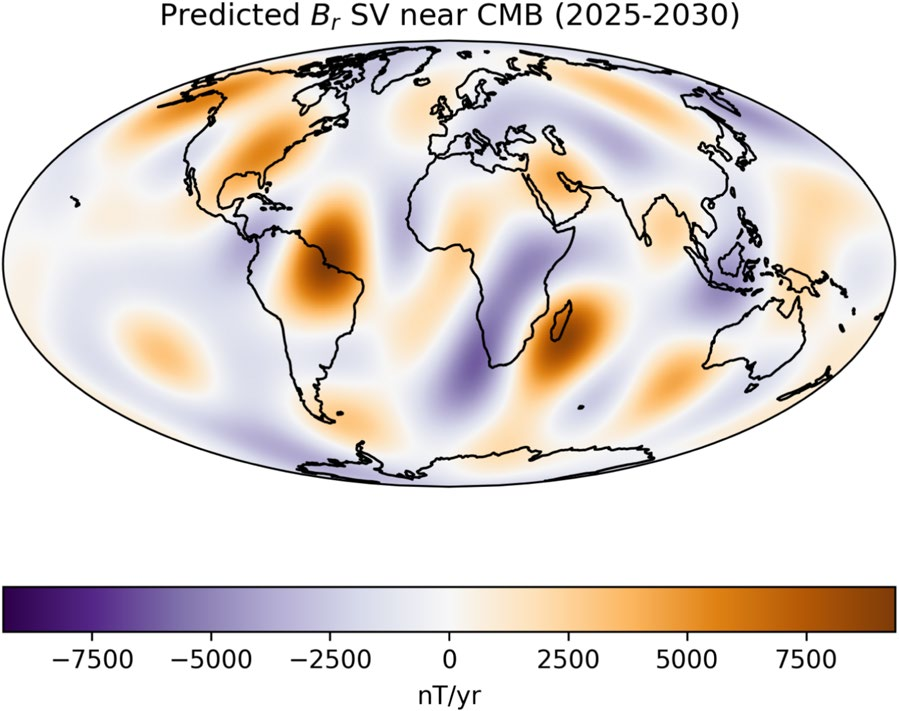
Mean predicted change in the radial magnetic field at the top of the geodynamo, truncated at degree $\ell \le 8$

Following the procedure outlined above, we produce a prediction for the mean SV between 2025–2030. The estimated core flow of the final smoother iteration between 2023.5$-$2024.5 is used to forecast the field in 2030, with the mean predicted change between 2030 and 2025 being reported as the SV candidate. The estimate of individual coefficient’s SV and associated uncertainties can be found in Table 1. Figure 7 shows the physical distribution of the mean predicted change in the form of the radial component of the magnetic field at the top of the outer core.

Some of the largest magnitude changes are expected in regions from under South America, the South Atlantic and Madagascar, corresponding to areas in and around the reverse flux patches associated with the South Atlantic Anomaly.

**Fig. 8:**
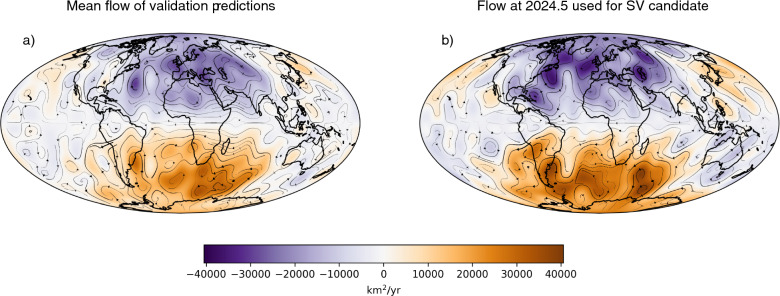
Toroidal scalar $\psi$ (color) and associated stream lines (arrows) for a the mean of the flows used in the eight validation forecasts and b the flow used in producing the 2025–2030 SV candidate. The plots show variations in the toroidal scalar of $\sim 8 \times 10^4$ km$^2$/yr over arcs of length $\sim 1$ rad. Therefore near the CMB of radius $\sim 3.5 \times 10^3$ km, we have velocities of $|\textbf{v}| \sim (8 \times 10^4 \text {km}^2/\text {yr})/(3.5 \times 10^3 \text {km}) \approx 23$ km/yr.

To examine the drivers of the predicted SV, we plot in Fig. 8, the core–surface flow used to forecast the field in 2030.

For comparison, we plot the mean of the eight flows used in the eight SV forecast validation experiments, i.e., the final (smoother) flow estimates for the periods 1978.5$-$1979.5, 1983.5$-$1984.5, 1988.5$-$1989.5, 1993.5$-$1994.5, 1998.5$-$1999.5, 2003.5$-$2004.5, 2008.5$-$2009.5, and 2013.5$-$2014.5. The typical velocity ($||\textbf{v}||$, computed as in Equation (15)) among the flows from the validation experiments is 20.95 km/yr, and for the flow of the SV candidate, $||\textbf{v}|| =$ 21.06 km/yr. Recall from Sect. 2.1 that the simple FF system does not model poloidal flow and therefore we make no direct inferences about dynamics below the surface. Instead, we highlight areas which may reflect consistency with previously inferred features of the interior dynamics. It is seen in the mean-flow estimate for the validation experiments, as well as the core flow used in producing the SV candidate, that the smoother suggests the existence of the eccentric gyre which is found in traditional flow inversions (see, e.g., Finlay et al. 2016; Gillet et al. 2015b; Baerenzung et al. 2018). As a result we find westward flow around the equator under a region spanning the Indian and Atlantic Ocean. In the average over time, as well as the recent flow estimate, the large-scale feature is the accumulation of several smaller vortices. In both figures, there is a symmetry about the equator in this pattern of vortices that suggests the presence of Taylor columns.

## Concluding remarks

We have used an EnKS with a simple frozen-flux model, to produce a candidate SV model for the period 2025–2030 (Table 1). The core field of the Kalmag field model, through degree $\ell =13$, was assimilated via an EnKF, every year, for ten years, into an ensemble of $N_e=512$ model runs. Smoothing (EnKS) was applied iteratively between assimilations (see Sects. 2.3.2, 3.1) and, despite the shortcomings of the simple FF model (see Sect. 2.1), the method was found to validate well against previous 5-year periods in which PGRFs were produced.

We noted in Sect. 2.2.1 that in the assimilation, the Kalmag core-field uncertainties were reduced by a factor of $1/\sqrt{\ell (\ell +1)}$, which ultimately resulted in better SV forecasts for past validation periods (see Fig. 4). This suggests the need to better understand the appropriate uncertainties for data assimilation/core-flow inversion with field models. It should be pointed out that this study neglects temporal correlations in the observation error which naturally leads to an overestimation of uncertainties. While there exist some works exploring methods for accounting for temporally correlated observation error in sequential filtering (see, e.g., Chang 2014; Raboudi et al. CorrNoise21), this needs to bee expanded upon and further explored in the context of geomagnetic DA. Additionally, while prescribed field model uncertainties may, in part, account for bias, such sources of uncertainty may be partially negated by the choice of scaling factors, such as $\mathcal {B}_\ell ^m$ of Equation (5). The use of the smoother also effectively gives weight to the observed change in Gauss coefficients (essentially average SV) over an assimilation period, which may be more well constrained. For this reason, we believe that future geomagnetic data assimilation work should also consider methods such as Variance Component Estimation (VCE, see, e.g., Kusche 2003a,2003b; Zhang and Lu 2021), for systematically determining optimal observation weighting.

Finally, we wish to emphasize again, the potential of the EnKS we have implemented, in other geomagnetic data assimilation systems. The method outlined in Sect. 2.3.2 can be implemented outside of an existing EnKF setup and requires no adjoint for the model. One only needs records of the prior and posterior ensembles at assimilation times. Because of this, the approach should be considered in any EnKF-based system, from those using full 3-D geodynamos (e.g., Gwirtz et al. 2024), to other, core–surface flow models (e.g., Huder et al. 2019).

**Table 1:** Table of the candidate, mean SV coefficients and uncertainties (in units of nT/yr)

$\ell$	m	$\dot{g}_\ell ^m$	$\dot{h}_\ell ^m$	$5\times \sigma _{\dot{g}_{\ell }^m}$	$5 \times \sigma _{\dot{h}_{\ell }^m}$
1	0	14.52	–	2.87	-
1	1	8.94	– 22.04	4.34	3.94
2	0	– 9.97	–	3.77	–
2	1	– 5.75	– 24.84	4.63	4.64
2	2	– 8.35	– 11.54	2.57	3.18
3	0	1.20	–	3.45	–
3	1	– 4.31	3.11	2.04	2.17
3	2	0.67	1.40	3.23	3.09
3	3	– 14.70	– 4.32	1.84	1.92
4	0	– 2.39	–	1.52	–
4	1	– 2.38	– 1.52	1.98	2.03
4	2	– 5.47	4.32	1.30	1.35
4	3	4.95	2.24	1.99	2.01
4	4	– 7.00	– 3.82	1.24	1.22
5	0	0.20	–	1.18	–
5	1	0.97	– 0.24	0.85	0.79
5	2	– 0.37	1.71	1.20	1.22
5	3	0.74	0.87	0.75	0.84
5	4	2.46	2.07	1.20	1.21
5	5	0.48	1.56	0.79	0.74
6	0	– 0.03	–	0.54	–
6	1	– 0.86	0.64	0.61	0.75
6	2	0.66	– 1.35	0.46	0.50
6	3	0.70	– 0.62	0.66	0.71
6	4	– 0.40	0.74	0.46	0.49
6	5	0.52	0.70	0.71	0.70
6	6	0.83	0.83	0.45	0.45
7	0	– 0.18	–	0.40	–
7	1	0.14	0.81	0.28	0.34
7	2	– 0.15	0.40	0.40	0.41
7	3	0.20	– 0.89	0.28	0.32
7	4	0.04	– 0.10	0.37	0.41
7	5	– 0.70	– 1.05	0.27	0.27
7	6	– 0.60	0.70	0.46	0.42
7	7	0.88	– 0.21	0.27	0.29
8	0	– 0.28	–	0.18	–
8	1	0.11	– 0.10	0.22	0.26
8	2	0.05	0.37	0.18	0.19
8	3	0.43	– 0.38	0.24	0.25
8	4	0.06	0.39	0.18	0.17
8	5	0.35	– 0.46	0.23	0.23
8	6	– 0.05	– 0.68	0.17	0.16
8	7	0.10	0.26	0.26	0.26
8	8	0.43	0.05	0.18	0.17

## Data Availability

All code used to produce and plot results is archived at 10.5281/zenodo.17210598.

## Appendix A: Data assimilation notation

See Table 2

**Table 2:** Table of notations used in the discussion of data assimilation methodology

$\textbf{y}_k$	Vector of magnetic field observations at time *k*
$\textbf{x}_k^\text {true}$	Unknown “true” state of the fluid flow and magnetic field at time *k*
$\textbf{H}$	Observation operator which relates the system state $\textbf{x}_k^\text {true}$ to the observations $\textbf{y}_k$
$\textbf{R}_k$	Observation error covariance at time *k*
$\varvec{\eta }_k$	Observation noise at time *k*
$N_e$	Number of ensemble members
$\textbf{K}_k$	Kalman gain at time *k*
$\varvec{\epsilon }_k^i$	Observation perturbation associated with ensemble member *i* as part of stochastic EnKF Houtekamer and Mitchell (1998)
$\textbf{P}_{k|K}$	Covariance for state estimate at time *k*, conditioned on data through time *K*
$\textbf{x}_{k|K}^i$	Ensemble member *i*, estimating the system state at time *k*, conditioned on observations through time *K*
$M_{k}^{T}$	Adjoint operator for time *k*
$\bar{\textbf{x}}_{k|K}$	Mean of ensemble members $\textbf{x}_{k|K}^i$
$\textbf{X}_{k|K}$	Array of all ensemble members, where the *ith* column is $\textbf{x}^i_{k|K}$
$\varvec{\mu }_{k|K}$	Array where each column is the ensemble mean $\bar{\textbf{x}}_{k|K}$

## Appendix B: Derivation of the ensemble approximation of the smoother

The derivation of the EnKS update formula in Equation (13) from the RTS tangent-linear form of Equation (11),

B.1 $${\mathbf{x}}_{{k|K}} = {\mathbf{x}}_{{k|k}} + {\mathbf{P}}_{{k|k}} {\mathbf{M}}_{k}^{T} T{\mathbf{P}}_{{k + 1|k}}^{{ - 1}} \left( {{\mathbf{x}}_{{k + 1|K}} - {\mathbf{x}}_{{k + 1|k}} } \right),{\text{ }}$$

is presented here. Let

B.2 $$\begin{aligned} \varvec{\Delta }\textbf{X}_{k+1|k}= & \textbf{X}_{k+1|k}-\varvec{\mu }_{k+1|k}, \end{aligned}$$

B.3 $$\begin{aligned} \varvec{\Delta }\textbf{X}_{k|k}= & \textbf{X}_{k|k}-\varvec{\mu }_{k|k}, \end{aligned}$$

be centered ensembles such that $\varvec{\Delta }\textbf{X}_{k+1|k}{\in }\mathcal {R}^{N_p\times N_e}$ and $\varvec{\Delta }\textbf{X}_{k|k}{\in }\mathcal {R}^{N_p\times N_e}$, where $N_p$ is the dimension of the state space and $N_p\gg N_e$. It follows that

B.4 $${\mathbf{P}}_{{k + 1|k}} = {\text{ }}\frac{1}{{N_{e} - 1}}{\text{ }}\Delta {\mathbf{X}}_{{k + 1|k}} {\text{ }}\Delta {\mathbf{X}}_{{k + 1|k}}^{T}$$

B.5 $${\mathbf{P}}_{{k|k}} = {\text{ }}\frac{1}{{N_{e} - 1}}{\text{ }}\Delta {\mathbf{X}}_{{k|k}} {\text{ }}\Delta {\mathbf{X}}_{{k|k}}^{T}$$

Now consider a state process

B.6 $$\begin{aligned} \textbf{x}_{k+1|k}=\textbf{m}(\textbf{x}_{k|k}), \end{aligned}$$

that is a nonlinear function of the current state space, such that

B.7 $$\begin{aligned} \textbf{dx}_{k+1|k}= & \left. {{\partial \textbf{m}\over \partial \textbf{x}}}\right| _{\textbf{x}=\varvec{\mu }_{k|k}} \textbf{dx}_{k|k}, \end{aligned}$$

B.8 $$\begin{aligned}= & \textbf{M}_k\;\textbf{dx}_{k|k}, \end{aligned}$$

where ${\mathbf{M}}_{k}^{T}$ is the adjoint operator seen in eq. B.1. As an alternative to performing the explicit partial differentiation indicated in Eq. B.7, one can instead define $\textbf{M}_k$ as a linear operator that maps ensemble deviations at time *k* to time $k{+}1$ such that

B.9 $$\begin{aligned} \varvec{\Delta }\textbf{X}_{k+1|k}=\textbf{M}_k \varvec{\Delta }\textbf{X}_{k|k}. \end{aligned}$$

Note, however, that both $\varvec{\Delta }\textbf{X}_{k+1|k}$ and $\varvec{\Delta }\textbf{X}_{k|k}$ have right-singular vectors ${1\over \sqrt{N_e}} \textbf{1}$ that correspond to a zero singular value. Therefore, the Singular Value Decompositions (SVD) of these matrices are:

B.10 $$\Delta {\mathbf{X}}_{{k + 1|k}} = {\text{ }}{\mathbf{U}}_{{k + 1|k}} {\mathbf{S}}_{{k + 1|k}} {\mathbf{V}}_{{k + 1|k}}^{T}$$

B.11 $$\Delta {\mathbf{X}}_{{k|k}} = {\text{ }}{\mathbf{U}}_{{k|k}} {\mathbf{S}}_{{k|k}} {\mathbf{V}}_{{k|k}}^{T}$$

where $\left\{ {\textbf{U}_{k+1|k}, \textbf{U}_{k|k}}\right\} {\in }\mathcal {R}^{N_p\times N_e-1}$ with orthonormal columns, $\left\{ {\textbf{S}_{k+1|k}, \textbf{S}_{k|k}}\right\} {\in }\mathcal {R}^{N_e-1\times N_e-1}$ are diagonal with positive entries, and $\left\{ {\textbf{V}_{k+1|k}, \textbf{V}_{k|k}}\right\} {\in }\mathcal {R}^{N_e\times N_e-1}$ with orthonormal columns. It now follows that

B.12 $${\mathbf{M}}_{k} = {\text{ }}\Delta {\mathbf{X}}_{{k + 1|k}} {\mathbf{V}}_{{k|k}} {\mathbf{S}}_{{k|k}}^{{ - 1}} {\mathbf{U}}_{{k|k}}^{T}$$

B.13 $$\begin{gathered} = \Delta {\mathbf{X}}_{{k + 1|k}} {\mathbf{V}}_{{k|k}} {\mathbf{S}}_{{k|k}}^{{ - 2}} {\mathbf{V}}_{{k|k}}^{T} {\mathbf{V}}_{{k|k}} {\mathbf{S}}_{{k|k}} {\mathbf{U}}_{{k|k}}^{T} \hfill \\ = \Delta {\mathbf{X}}_{{k + 1|k}} \left( {\Delta {\mathbf{X}}_{{k|k}}^{T} T\Delta {\mathbf{X}}_{{k|k}} } \right)^{ + } \Delta {\mathbf{X}}_{{k|k}}^{T} \hfill \\ \end{gathered}$$

where the superscript “+" indicates a pseudo-inverse. The pseudo-inverse is a generalization of the traditional inverse such that for an arbitrary matrix $\textbf{A}$, its pseudo-inverse $\textbf{A}^{+}$ is such that

B.14 $$\begin{aligned} \textbf{E}_1= & \textbf{A} \textbf{A}^{+}, \end{aligned}$$

B.15 $$\begin{aligned} \textbf{E}_2= & \textbf{A}^{+} \textbf{A}, \end{aligned}$$

where $\textbf{E}_1$ and $\textbf{E}_2$ are projection matrices, which is a generalization of the identity matrix.

Next, consider the matrix $\textbf{P}_{k+1|k}$ in Eq. B.1 whose inverse is sought, but note from the SVD of $\varvec{\Delta }\textbf{X}_{k+1|k}$ in Eq. B.10 that

B.16 $${\mathbf{P}}_{{k + 1|k}} = \frac{1}{{N_{e} - 1}}{\mathbf{U}}_{{k + 1|k}} {\mathbf{S}}_{{k + 1|k}}^{2} {\mathbf{U}}_{{k + 1|k}}^{T}$$

Thus, even though $\textbf{P}_{k+1|k}{\in }\mathcal {R}^{N_p\times N_p}$ it only has a rank of $N_e{-}1$, and so a formal inverse does not exist. However, the pseudo-inverse can be seen to be

B.17 $$\begin{aligned} {\mathbf{P}}_{{k + 1|k}}^{ + } & = (N_{e} - 1){\mathbf{U}}_{{k + 1|k}} {\mathbf{S}}_{{k + 1|k}}^{{ - 2}} {\mathbf{U}}_{{k + 1|k}}^{T} \\ & = (N_{e} - 1){\mathbf{U}}_{{k + 1|k}} {\mathbf{S}}_{{k + 1|k}} {\mathbf{V}}_{{k + 1|k}}^{T} {\mathbf{V}}_{{k + 1|k}} {\mathbf{S}}_{{k + 1|k}}^{{ - 4}} {\mathbf{V}}_{{k + 1|k}}^{T} {\mathbf{V}}_{{k + 1|k}} {\mathbf{S}}_{{k + 1|k}} {\mathbf{U}}_{{k + 1|k}}^{T} \\ & = (N_{e} - 1)\Delta {\mathbf{X}}_{{k + 1|k}} \left( {\Delta {\mathbf{X}}_{{k + 1|k}}^{T} \Delta {\mathbf{X}}_{{k + 1|k}} } \right)^{{ + 2}} \Delta {\mathbf{X}}_{{k + 1|k}}^{T} \\ \end{aligned}$$

such that

B.18 $${\mathbf{P}}_{{k + 1|k}}^{ + } {\mathbf{P}}_{{k + 1|k}} = {\mathbf{P}}_{{k + 1|k}} {\mathbf{P}}_{{k + 1|k}}^{ + } = {\mathbf{U}}_{{k + 1|k}} {\mathbf{U}}_{{k + 1|k}}^{T} = {\mathbf{E}}.{\text{ }}$$

where $\textbf{E}$ is a projection matrix.

Finally, substituting eqs. B.5, B.13 and B.17 into the ensemble version of Eq. B.1 yields:

B.19 $${\mathbf{X}}_{{k|K}} = {\mathbf{X}}_{{k|k}} + {\mathbf{P}}_{{k|k}} {\mathbf{M}}_{k}^{T} {\mathbf{P}}_{{k + 1|k}}^{{ - 1}} \left( {{\mathbf{X}}_{{k + 1|K}} - {\mathbf{X}}_{{k + 1|k}} } \right), = {\mathbf{X}}_{{k|k}} + \left[ {\frac{1}{{N_{e} - 1}}\Delta {\mathbf{X}}_{{k|k}} \Delta {\mathbf{X}}_{{k|k}}^{T} } \right]\left[ {\Delta {\mathbf{X}}_{{k|k}} \left( {\Delta {\mathbf{X}}_{{k|k}}^{T} \Delta {\mathbf{X}}_{{k|k}} } \right)^{ + } \Delta {\mathbf{X}}_{{k + 1|k}}^{T} } \right]\,\left[ {\left( {N_{e} - 1} \right)\Delta {\mathbf{X}}_{{k + 1|K}} \left( {\Delta {\mathbf{X}}_{{k + 1|k}}^{T} \Delta {\mathbf{X}}_{{k + 1|k}} } \right)^{{ + 2}} \Delta {\mathbf{X}}_{{k + 1|k}}^{T} } \right]\left( {{\mathbf{X}}_{{k + 1|K}} - {\mathbf{X}}_{{k + 1|K}} } \right),$$

B.20 $$\Delta {\mathbf{X}}_{{k + 1|k}}^{ + } = \left( {\Delta {\mathbf{X}}_{{k + 1|k}}^{T} \Delta {\mathbf{X}}_{{k + 1|k}} } \right)^{ + } {\text{ }}\Delta {\mathbf{X}}_{{k + 1|k}}^{T}$$

which is Equation (13), where

B.21 $$\Delta {\mathbf{X}}_{{k + 1|k}}^{ + } = \left( {\Delta {\mathbf{X}}_{{k + 1|k}}^{T} \Delta {\mathbf{X}}_{{k + 1|k}} } \right)^{ + } {\text{ }}\Delta {\mathbf{X}}_{{k + 1|k}}^{T}$$
